# Characteristics of Wild Boar-Damaged Farmland and Assessment of Effectiveness of Prevention Measures in Northeast China

**DOI:** 10.3390/ani14213079

**Published:** 2024-10-25

**Authors:** Ning Zhang, Yang Hong, Xiaoqing Yuan, Liulisha Zhao, Minghai Zhang

**Affiliations:** College of Wildlife and Protected Area, Northeast Forestry University, Harbin 150040, China; 18846797329@163.com (N.Z.); hy1624@126.com (Y.H.); 18227924605@163.com (X.Y.); 18845789946@163.com (L.Z.)

**Keywords:** wild boar *(Sus scrofa*), human–wildlife conflict, farmland damage, prevention measures

## Abstract

Although wild boar invasion is a common human–wildlife conflict worldwide, it is difficult to solve. Understanding the characteristics of wild boar invasion into farmland, accurately predicting the areas where wild boar invasions will occur, and implementing effective prevention and control measures are key to effectively alleviating conflicts between humans and wild boars. From 2021 to 2023, we conducted field surveys and experimentally determined the effectiveness of prevention and control measures in the Huanan and Tonghe Counties in order to explore the landscape and farmland factors affecting wild boar invasion, as well as to evaluate the effectiveness of different combinations of prevention and control measures. The results indicate that the mean patch area, patch area coefficient of variation, and forest type edge density show positive linear correlations with the invaded area, which is positively associated with the presence of ditches and negatively correlated with the existence of electronic fences. Meanwhile, there are non-linear relationships between invasion and the edge density of farmland type, the proportion of forests, the Shannon uniformity index, the proportion of residential areas, the distance from the village, the distance from the forest, and the population density. In particular, the risk of wild boar invasion is higher in the border area between forest and farmland. The loss rate of farmland under the different control measures (compound) was significantly lower than that in the electronic fence group (single) (*p* < 0.05). Based on these findings, we put forward the following suggestions for the prevention and control of wild boar: (1) widen and deepen the narrow landscape elements around farmland; (2) focus on the border area between forest and farmland; and (3) use compound prevention and control measures. This study provides a comprehensive foundation for future wild boar prevention and control research, and it contributes to the formulation of effective wild boar prevention and control policies worldwide.

## Simple Summary

Considering the global problem of wild boar invasion, we thoroughly analyzed the factors affecting wild boar invasion through detailed field investigations and scientific experiments, as well as evaluating the effectiveness of different prevention and control strategies. This study reveals the significant effects of landscape structure and farmland characteristics on wild boar behavior patterns, additionally emphasizing the superiority of integrated control measures such as electronic fencing. We propose that optimizing the landscape layout, strengthening the monitoring of key areas, and adopting comprehensive prevention and control measures may effectively mitigate the hazard. This study not only provides a scientific basis for the management of wild boars but also serves as an important reference for the formulation of global wild boar prevention and control strategies.

## 1. Introduction

In recent decades, the conflict between people and wild boars has become increasingly fierce, mainly reflected in the damage to crops caused by wild boars and large economic losses. The conflict between human beings and wild animals reflects the contradiction between human beings and symbiotic wild animals, but, in essence, it is a dangerous signal sent by nature regarding whether human beings can live in harmony with the ecological environment. How to view and resolve this conflict is related to the protection of the ecological environment and biodiversity, and its severity and complexity have become a central issue in wildlife protection and management [1]. The damage in Europe doubles every 10 years, and, at present, damage caused by wild boars incurs costs of about EUR 80 million per year [2,3]. In 2016, wild boars caused about USD 190 million in damage to six crops in 11 states and caused up to USD 1.5 billion in crop damage and containment costs across the United States [4,5]. Wild boar are known to invade fields in around 70% of provinces in China; for example, in the Hunchun area of Jilin Province, 1076 wild boar hazard incidents occurred from 2012 to 2013 alone, with a hazard area of 518.8 hm^2^, causing economic losses of about CNY 3.632 million [6]. Therefore, alleviating the conflict between humans and wild boars has become a worldwide problem. Wild boar cause damage such as crop damage, orchard damage, pasture and grassland damage, horticultural plant damage, forestry damage, land degradation, and infrastructure damage, as well as threats to personal safety. This damage not only causes direct economic losses but may also cause a change in the ecological balance and uncertainty of agricultural production.

The invasion of wild boars into farmland is influenced by various factors, such as the type and growth stage of crops, landscape factors, and basic characteristic factors of the invaded farmland (hereinafter referred to as “farmland factors”), among others. Most studies believe that wild boar prefer digestible and nutritious crops [7,8], and, as the crops mature, wild boars show a high preference for farmland [9]. While crop species and growth stage have widely been considered in wild boar invasion research, how landscape and farmland factors influence wild boar invasions have scarcely been considered in the global literature, and the common characteristics of wild boar invasions have not yet been summarized. For example, taking the distance from the forest as an influencing factor, some researchers believe that it is negatively linearly correlated with wild boar invasion [10,11], while others believe that the two do not share a linear relationship [12]. Therefore, it is necessary to carry out further research in this area with respect to various regions, improve the global research on wild boar invasions, and improve the prediction of high-risk areas of wild boar invasion, such that effective prevention and control measures can be formulated in a timely manner.

There are two types of prevention and control measures for wild boar invasions: lethal and non-lethal. Lethal measures reduce the harm caused by reducing the quantity and changing the age structure of wild boars, mainly through methods such as hunting, poisoning, and trapping, which have been proven to be effective in various different countries [13,14,15]. Non-lethal prevention and control measures do not change the population characteristics of the wild boar, but achieve the purpose of the prevention and control of wild boar through affecting their behavioral characteristics, mainly including the use of distractors, shelters, sound and light interference, and other technologies, which are suitable for areas that lack professional hunting teams, where the number of wild boar does not reach the hunting level, or where hunting is prohibited [16]. Electronic fences are an effective non-lethal prevention and control measure [17,18,19], but wild boars may quickly adapt to a single measure. Therefore, we evaluated the composite effect of combining electronic fences with other measures, compared to a single control group using only electronic fences, and calculated the cost-effectiveness ratio. In this way, we aim to provide a reference for formulating reasonable and effective wild boar prevention and control programs.

The key to the effectiveness of wild boar prevention and control lies in the successful prediction of wild boar invasion areas and the implementation of effective prevention and control measures. We propose the following hypotheses: (1) there are linear or non-linear relationships between wild boar invasions and various landscape and farmland factors; (2) wild boar invasion occurs in the border area between farmland and forest; and (3) compound prevention and control measures are better than single-type prevention and control measures.

## 2. Study Area and Methods

### 2.1. Study Area

**Figure 1:**
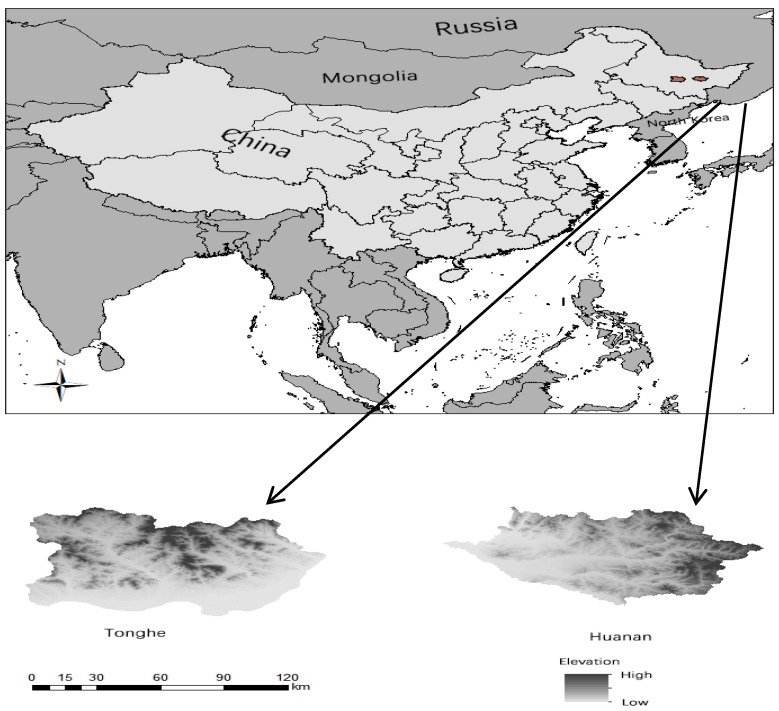
Schematic representation of the location of the study area.

This study was conducted in the Huanan and Tonghe Counties in Heilongjiang Province, China. Huanan County (129°54′55′~131°16′2′ E, 46°22′23′~46°20′15′ N) is located in the eastern part of Heilongjiang Province, with a total area of 4417.9 km^2^ [20]. Tonghe County (128°8′54′~129°25′31′ E, 46°4′58′~46°17′16′ N) is located in the central part of Heilongjiang Province with a total area of 5678 km^2^ [21] (Figure 1). The wild plants in the two counties are mainly Korean pine (*Pinus koraiensis*), accompanied by a variety of cold temperate and temperate tree species, such as smelly fir (*Abies nephrolepis*), Chinese ash (*Juglans mandshurica*), and water-curved willow (*Fraxinus mandschurica*). Wild animals include the Siberian tiger (*Panthera tigris*), black bear (*Ursus thibetanus*), and wild boar (*Sus scrofa*).

Huanan County has 10 townships under its jurisdiction, while Tonghe County has 8 townships under its jurisdiction. By 2020, the resident population of Huang County was about 287,000. Ghetto County has a permanent population of about 180,000, of which 70% comprises the agricultural population. In addition, Tonghe County is also a settlement of hui, Mongolian, Korean, and other ethnic minorities. Agriculture is one of the main economic sources for the residents of the two counties. According to the statistics, Huanan County alone has 3.425 million mu of cultivated land. In 2018, Huanan County planted 1.563 million mu of corn, 900,000 mu of soybean, 801,000 mu of rice, and 157,000 mu of miscellaneous grains, beans, and cash crops.

### 2.2. Site and Area of Wild Boar Invasion

We conducted a survey focused on damaged farmland using a sampling method. According to the information provided by the County Forestry and Grassland Bureau, under the leadership of local farmers, a field investigation was conducted using damaged farmland as a sample square, mainly checking the traces of wild boar activities on the sample farmland (e.g., lying down, feces, footprints, and feeding traces) in order to confirm the invasion of wild boars. At the same time, detailed information such as the sample point (the center point of the damaged farmland) and the area of the sample farmland were recorded. From August 2021 to October 2022, a total of 116 destroyed farmlands were established in 6 forest farms and 21 villages in Huainan County and Tonghe County, Heilongjiang Province.

### 2.3. Landscape Factors

The used land cover data were derived from the results of the classification of land use in China based on Landsat SR remote sensing data in 2021 by Yang Jie et al. [22]. According to the actual land cover types in Huanan County and Tonghe County, we divided the landscape types into seven categories, namely farmland, forest, shrub, shrubbery, grassland, residential areas, and water and other land. The spatial resolution of the grid data for the land cover classification was 30 m × 30 m. A 1.25 km wide buffer was centered on the Arc GIS10.3 and Fragstats 4.2.1 software [23], and nine landscape characteristic factors within the buffer were considered; namely, forest percentage (FPLAND), water percentage (WPLAND), percentage of residential area (RPLAND), mean patch area (AREA_MN), the patch area coefficient of variation (AREEA_CV), the Shannon diversity index (SHDI Shannon), Shannon evenness index (SHEI Shannon), forest type edge density (FED), and farmland type edge density (LED).

### 2.4. Farmland Factors

A total of 15 fields were obtained through field investigations, for which ArcGIS was used for the extraction of factors, including the distance from the center of the forest (DISWOOD), the distance from the village (DISCYS), the distance from the road (DISROD), the distance from the river (DISRIV), elevation (ELE), slope (TRASP), slope direction (SLP), crop height (HC), crop cover (CC), the watercourse (CANAL), electronic fence (EF), number of prevention and control measures (PM), population density (PD), GDP per capita (GDP), and distance from the original starting point of the wild boar (OSP); for the latter, a 3 km wide area from the center of the wild boar invasion area was considered, and the reverse tracking of wild boar footprints in this area was carried out. The farthest trace disappearance point from the center of the invaded area was used as the original starting point of the wild boar.

### 2.5. Spatial Distribution Data Acquisition

To further understand the spatial distribution characteristics of wild boar invasion, we chose Huanan County in the study area as the research object to predict the spatial distribution of wild boar invasions. We conducted a supplementary survey in 2023, based on the survey data collected in 2021–2022. During the two surveys, 105 wild boar invasion sites were collected in Huanan County. After deleting sites less than 1 km using SDM Tools, 33 wild boar invasion sites were finally retained. According to the actual situation of Huanan County, we chose 17 environmental variables related to wild boar fields, divided into terrain, landscape, and human factors as the three main categories, specifically including altitude, slope, slope direction, distance from settlements, road density, river density, forest percentage, the percentage of farmland, distance from river, distance from coniferous forest, distance from road, farmland fragmentation, Shannon diversity index, broadleaf forest proportion, coniferous forest proportion, and mixed forest proportion.

### 2.6. Sample Setting of Prevention and Control Measures

We conducted a study to evaluate the effectiveness of wild boar prevention and control measures in Huainan County from August to October 2022. The study area was divided into 12 groups with 3 plots, each with an area of 0.5 hm^2^, for a total of 36 sample plots (Table 1). These plots were all selected in corn fields at the edge of forests, where wild boars pose a serious threat. The experiment combined solar electronic fences with various other prevention and control measures, which were compared with groups using electronic fences alone and without any prevention and control measures, in order to evaluate the prevention and control effects of each group. The prevention and control measures included using loudspeakers to play human shouting, banging gongs, and dog barking, as well as using a mixture of yellow sulfur powder and lime powder or peppermint oil, chili oil, and lard as a repellent. In addition, red flashing solar warning lights with different heights were set up. The implementation of these measures started at the beginning of the study and ended when harm was caused by wild boars, with an observation period of 50 days. Every morning, it was checked whether the corn in each plot had been eaten or trampled by wild boars, and whether the prevention and control measures had been destroyed by wild boars. The area of corn samples, the number of wild boar invasions, and the duration of harm during the study period were calculated, allowing the prevention and control costs for each group during the study period to be obtained. The loss rate of wild boar damage was defined as the percentage of the total area of corn destroyed by wild boars from the beginning of the experiment to the harvest period of the crops. Therefore, it was necessary to investigate the average yield of autumn-harvested corn and the average purchase price in the local market that year.

### 2.7. Data Processing

#### 2.7.1. The Relationships Between Landscape Factors, Farmland Factors, and Wild Boar Invasion Area

This study used the generalized additive model (GAM) to explore the relationships between the landscape factors, farmland factors, and wild boar invasion area. The GAM establishes the relationship between an explanatory variable and a response variable through a connection function. The GAM performs both linear and smooth function fitting for the explanatory variables, which is suitable for the case where both linear and non-linear relationships may exist between the explanatory and response variables. The basic form of the GAM is as follows:

g(μ) = α + f1(X1) + f2(X2) + … + fp(Xp)

The GAM includes three parts: the explanatory variable Xp and the response variable Y, the connection function g(μ), and the smoothing function fp(Xp). Here, μ = E(Y~X1, X2, …, Xp) is the mathematical expectation of the response variable Y, and α is the intercept. The form of the connection function g(μ) is determined by the distribution of the response variables, and different connection functions correspond to different distributions.

Collinearity was tested using the Pearson correlation coefficient (r), and variables with |r| > 0.8 were removed [24]. The connection function was selected through an exploratory analysis of the response variables using distribution tests. In the construction of the GAM model, the forward selection method was used to gradually add linear or non-linear terms. The optimal model was selected according to the AIC (Akaike Information Criterion) and generalized cross-validation (GCV) value, and the model with minimum GCV and AIC values was judged as the optimal model. This was implemented using the “mgcv” package in R3.6.1. The significance level of the difference was 0.05.

#### 2.7.2. Spatial Distribution Model

The MaxEnt Model (maximum entropy model) has been applied in many ecological fields, such as species habitat suitability evaluation, disease and insect pest prediction, and forest fire line prediction [25,26,27]. The MaxEnt model was used to predict the risk areas of wildlife harm to crops, allowing for the implementation of corresponding prevention and control measures to reduce the damage, thus alleviating the conflict between human and wildlife. In this way, a wild boar risk distribution model for Huanan County was constructed, and the average value of 10 simulations was taken as the result. The model of environmental data importance was analyzed through a Jackknife test and the accuracy was evaluated according to the area under the ROC curve (AUC). Through model construction, the wild boar hazard probability distribution map for farmland in Huanan County was obtained. In this map, the probability value ranged between 0 and 1, where a larger value indicated a higher probability of wild boars invading the farmland. Therefore, the probability distribution map was divided into three risk levels, with 0.0–0.1 indicating a low risk, 0.1–0.4 indicating a medium risk, and 0.4–1.0 indicating a high risk.

#### 2.7.3. Analysis of the Effectiveness of Prevention and Control

The Mann–Whitney U-test was used to determine the variability in the combined effects of the various control measures. When *p* < 0.05, the difference was considered significant. The cost–benefit ratio with respect to each set of prevention and control measures was calculated as the ratio of benefit to the total cost of prevention and control. Here, benefit is defined as the benefit obtained from corn when protecting it against the invasion of wild boars through the use of the prevention and control measures. Meanwhile, the total cost of prevention and control is defined as the patrolling costs plus those of the prevention and control measures.

$$BCR=\frac{B}{C}=\frac{S\times (1-G)\times R}{P+M},$$

where *BCR* is the cost–benefit ratio of prevention and control, *B* denotes the benefit (CNY), *C* is the total cost of prevention and control (CNY), *S* is the site area (hectares), *G* is the crop loss rate (%), *R* is the crop price (CNY/hectare), *P* is the cost of prevention and control measures (CNY), and *M* is the patrol cost.

## 3. Results

### 3.1. Landscape Characteristics of the Affected Farmland

**Figure 2:**
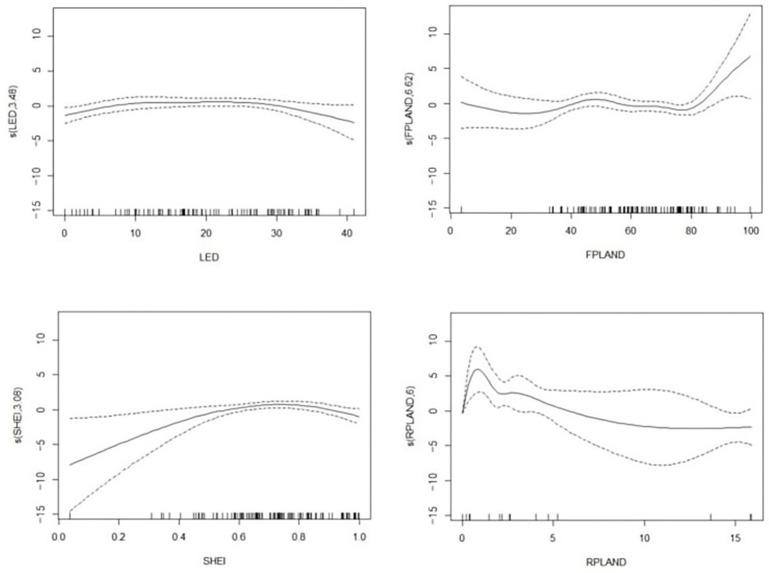
Non-linear functional relationships between area of destruction and four explanatory variables: LED, edge density of farmland; FPLAND, percentage of forest; SHEI, Shannon evenness index; and RPLAND, percentage of residential area. The dashed line in the figure represents the threshold interval.

The mean patch area, patch area coefficient of variation, and forest type edge density showed linear and positive correlations with the invaded area. Furthermore, there were clear non-linear relationships between the farmland area and edge density, forest proportion, the Shannon evenness index, and the proportion of residential areas (Table 2). The farmland edge density curve turns at 27, rising slowly before this value and decreasing above it. The forest proportion and Shannon evenness index curves increased overall. The proportion of residential areas curve showed a downward trend, with a higher proportion of residential areas leading to a smaller invaded area (Figure 2).

### 3.2. Basic Characteristics of Damaged Farmland

**Figure 3:**
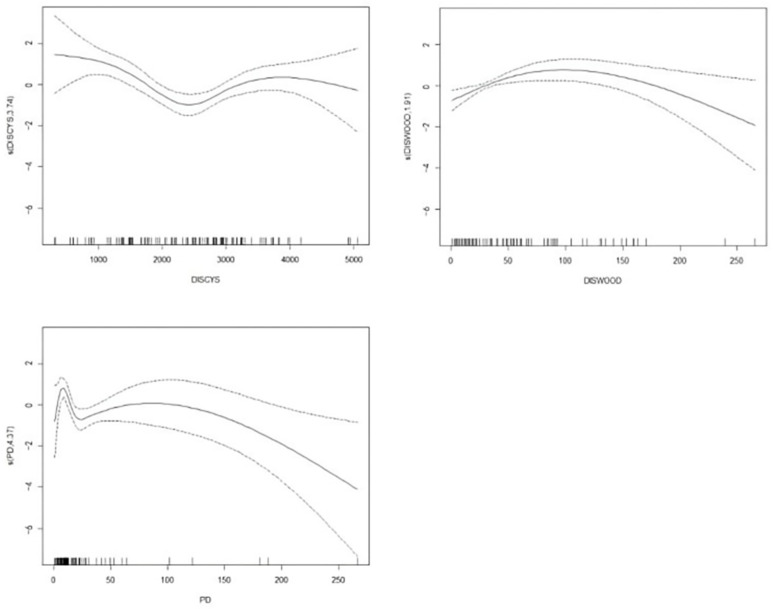
Non-linear functional relationships between the invaded field area and the three farmland factors. The x-axis represents the environmental variables, the y-axis represents the non-linear functions, the solid line represents the specific model fitted, and the dashed line represents the degrees of freedom used to constrain the smoothness of the model.

The area of invaded fields was positively correlated with the presence of ditches and negatively correlated with the presence of electronic fences, the distance from the village, the distance from the forest, and population density (Table 3). The distance from the village curve turns at 2300 m, decreasing slightly below 2300 m and rising slowly above 2300 m. The distance from the forest curve turns at 100 m, rising below 100 m, then decreasing when it is greater than 100 m. The population density curve showed an overall downward trend; that is, with increasing population density, the invaded area decreases (Figure 3). Therefore, Hypothesis 1 was supported.

### 3.3. Spatial Distribution Prediction

**Figure 4:**
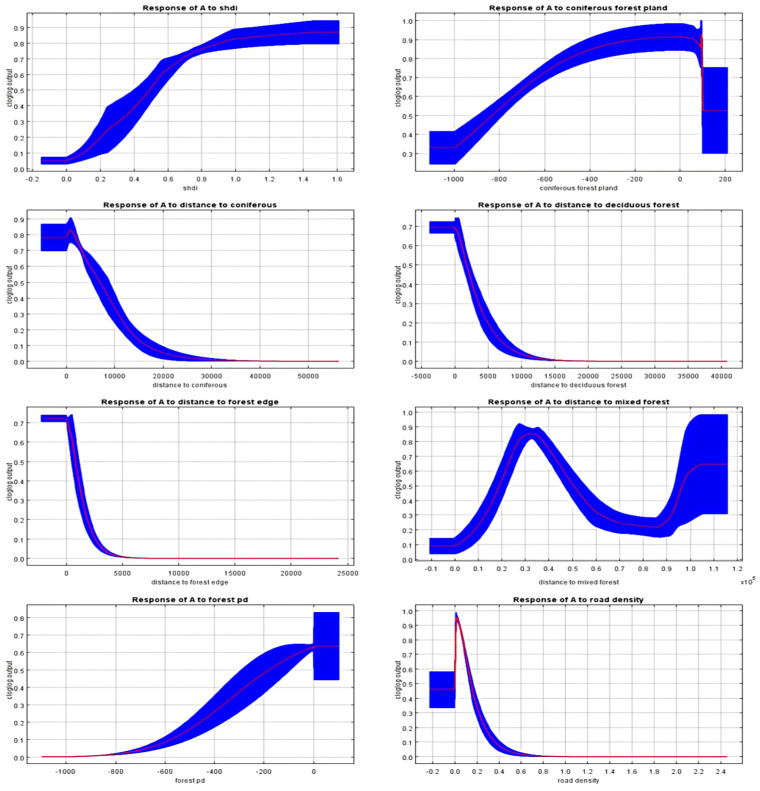
Response curves of environmental variables to predicted probability. The blue area represents the threshold, and the red line response curves of environmental variables to predicted probability.

**Figure 5:**
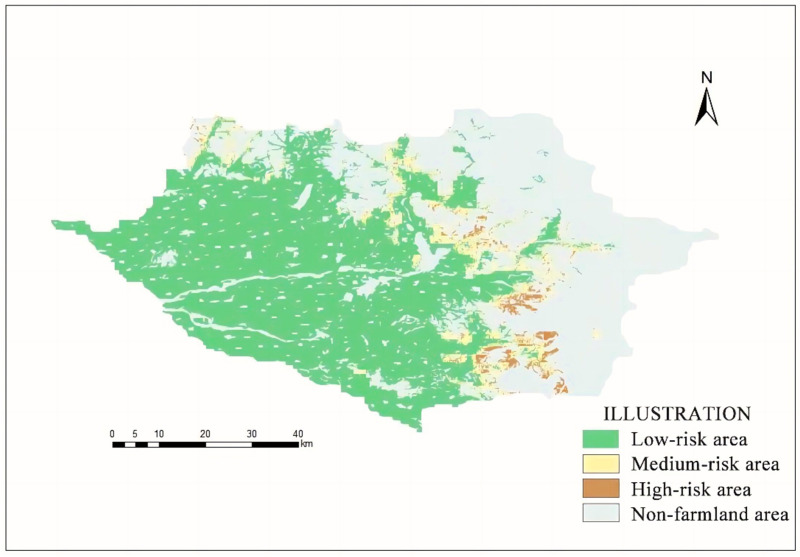
Wild boar invasion risk distribution diagram for Huanan County.

The MaxEnt Model was repeated for 10 runs, and the average AUC was 0.973. Among all environmental factors, the distance from the coniferous and broad-leaved mixed forest contributed the highest, reaching 14.2%, followed by patch density (13.0%), forest density (11.2%), distance from coniferous forest (11.1%), distance from the forest margin (9.0%), road density (8.3%), the Shannon diversity index (6.7%), and distance from broad-leaved forest (5.9%). The contribution rates of the other environmental factors were less than 5%. The sensitivity analysis of the effects of various environmental variables on the distribution of wild boar hazards revealed that wild boar hazards occurred between 200 and 300 m from the including altitude, and the risk index was maximized when the slope was within 0–10° or 50–150°. Regarding the distance from the forest margin, the distance from the coniferous forest, and the distance from the broad-leaved forest, the risk index decreased significantly as the values of these environmental factors increased. Furthermore, the risk index decreased as road density and river density increased. With a decrease in the coniferous forest proportion, the proportion of broad-leaved forest, and mixed forest proportion, the risk index also decreased. With an increasing patch density, fragmentation, and diversity index, the risk index increased. At about 5000 m away from settlements, the risk index reached a maximum. Meanwhile, the risk was nearly 0 at 5000 m from the forest margin (Figure 4). The high-, medium-, and low-risk areas of wild boar invasion were 128.11 hm^2^, 458.68 hm^2^, and 4093.99 hm^2^, respectively. The high-risk areas were mainly focused at the junction of forest and farmland in the eastern and southern parts of the study area (Figure 5). Therefore, Hypothesis 2 was supported.

### 3.4. Evaluation of the Prevention and Control Effect

Data processing was conducted using the IBM SPSS27 and Excel 2016 software, and a Mann–Whitney U-test was used to evaluate the significance of the differences in effectiveness between different prevention and control measures. When the *p* value was less than 0.05, the difference was considered significant. Through a regression fitting analysis, we evaluated the relationship between the corn loss rate and the invasion frequency of wild boars, additionally calculating the R^2^ and F values of the model. In addition, the cost–benefit ratio of each prevention and control measure was calculated (i.e., the ratio of the benefits obtained from protecting corn through prevention and control measures to the total cost of prevention and control). The results indicated that, in the non-control group (L), the number of invasions (4.67 ± 1.53), the number of wild boars (12.67 ± 4.04), and the loss rate (54 ± 15) were the highest. The loss rates in groups A–J and L (with and without prevention and control measures, respectively) were significantly different (*p* < 0.05), and the existence of prevention and control measures effectively hindered wild boar invasions. Of the groups with prevention and control measures, group F suffered the most invasions; while group E and group H were invaded the least, followed by group K. In particular, the loss rate of group H was significantly lower than that of group K (*p* < 0.05). Therefore, the protective effect of an electronic fence in combination with other prevention and control measures was better than that of the electronic fence alone. We compared the groups, superimposing different protective measures (i.e., groups A–J) with each other, and found no significant difference between these groups (*p* > 0.05; Table 4). Therefore, Hypothesis 3 was supported.

### 3.5. Cost-Effectiveness of Prevention and Control

According to the statistics of the Huanan County Forestry and Grassland Bureau, the average local trading price of corn and other major crops was 9500 CNY/hm^2^. During the study period, the patrol cost was set at CNY 200, according to the local economic level. The prevention and control costs (CNY) were as follows: Group A, 1232; Group B, 1232; Group C, 1341; Group D, 1265; Group E; Group F, 1373; Group G, 1406; Group H, 16,416; Group I, 1316; Group J, 1297; and Group K, 1200.

Through calculation, the cost-benefit ratio of wild boar harm to farmland prevention and control is highest in Group B, with a ratio of 3.28; then follow Group A with a ratio of 3.25, Group I with a ratio of 3.10, Group D with a ratio of 3.04, Group E with a ratio of 2.99, Group F with a ratio of 2.98, Group H with a ratio of 2.91, Group C with a ratio of 2.76, and Group G with a ratio of 2.95. Group J has the lowest cost–benefit ratio for prevention and control, with a ratio of 2.63. The cost–benefit ratio of the K group using only electronic fences for prevention and control is 1.56.

## 4. Discussion

A landscape is a collection of various ecosystems. The wild boars that harm farmland often move in between forest and farmland ecosystems. To study the characteristics of wild boar invasion, we need to conduct analyses from a landscape-based perspective [11,28]. We found significant positive linear correlations between mean patch area, the patch area coefficient of variation, and forest type edge density with invaded field area. The average patch area reflects the degree of landscape fragmentation, and at the landscape scale, the larger the average patch area, the lower the degree of fragmentation. Safety is one of the key factors for the survival of wildlife [29], and the lower the degree of fragmentation, the larger the area of continuous forest or farmland, allowing for the more effective concealment of wild boars. This increases the likelihood that they will invade the adjacent farmland and feed on crops, and so, the area of farmland destroyed by wild boars increases. The patch area coefficient of variation represents the degree of dispersion of the landscape, where a larger value indicates a greater difference between patch areas and a higher diversity of the landscape. When the landscape diversity is high, wild boars can obtain more abundant food sources, which helps to ensure the maintenance and growth of their population. As the population of wild boars increases, their invasion area into farmland increases accordingly. At the same time, with a higher edge density of forest types and longer forest boundaries, the larger is the contact area between wild boars and other land types (e.g., farmland) through these boundaries, making it more likely for them to invade farmland while remaining harder to detect; even if they are detected, they can quickly escape. The result is that a higher edge density of forest types leads to a larger area of farmland invaded by wild boars.

Narrow landscape elements, such as ditches, are an important ecological corridor for wildlife. As a typical channel and activity area for many species, it can provide a certain degree of concealment in human-dominated landscapes [30]. In this study, invasion by wild boars was positively associated with the presence of ditches. Ditches can be divided into artificial and natural ditches: artificial ditches are distributed around farmland, and one of their roles is to resist the invasion of wild boars into fields; however, if the depth of the ditch is shallow, and its slopes are smooth enough, wild boar can easily cross them. Furthermore, in contrast to their intended deterring effect, as ditches can provide water and mud bath conditions, they may attract wild boars. Natural ditches are potential channels for wild boar to pass from the forest into farmland. Therefore, areas with ditches had larger areas of wild boar invasion.

Forests can provide good hiding conditions for wild boar. Many studies have stated that the distance from the forest is negatively linearly correlated with the degree of invasion; that is, the closer the forest, the more serious the wild boar invasion area [10,31]. However, this study found that wild boars invaded the field most seriously about 100 m from the forest, maintaining a certain invasion intensity within 0–100 m. After 100 m, the area of the invaded field decreased with distance from the forest (Figure 3). We speculate that this may be related to the quality of crops and the feeding strategy of wild boars. Crops within 100 m from the forest are affected by the forest, thus having limited light, limited nutrition, and may suffer from more serious diseases and pests, slightly lowering the quality of these crops. Meanwhile, crops 100 m away from the forest have more sufficient light and less nutritional competition with wild plants, leading to higher-quality crops. For the efficient acquisition of plants, high-quality crops are preferred for feeding. However, considering factors such as safety and the ability to rapidly escape, wild boars will not move too far away from the forest when invading the field, reflecting that the distance between forest edges is the main factor affecting the harm of wild boars to farmland.

In this study, wild boar invasion was mainly concentrated in the boundary area between farmland and forest, similar to the results of Jiang Xiaoping et al. (2017) [32]. The junction area between farmland and forest has the following characteristics: first, it is close to the forest, meaning that the wild boar is not easily found when invading the field and can escape quickly after invading the field; second, it is often far away from the human activity area, making is difficult to conduct real-time supervision. This, once again, proves that the distances from the forest and human activity are important factors affecting the wild boar invasion area. In future wild boar prevention and control work, efforts should be made to optimize the land use pattern or change the landscape characteristics of the agricultural and forestry border area significantly, in order to reduce the area of wild boar invasion and alleviate conflicts between humans and wild boars.

The strong adaptability of wild boar is one of the reasons for the difficulty regarding their prevention and control. Effective prevention and control measures are based on the wild boar’s fear of new things. When the wild boar finds that there is no harm to their safety, it will quickly adapt to these prevention and control measures, leading to a decline in the prevention and control effect. During the field investigation, we found that the effects of prevention and control measures such as solar warning lamps, loudspeakers, and repellents alone were not good, and wild boar would still frequently enter the farmland to eat crops. Schlageter et al. assessed that the effectiveness of solar flashing lights in preventing and controlling wild boar damage to farmland was only 8.1% [33]. However, this study found that the loss rates in groups A-J with different control measures were significantly different from those in group K, using only a single measure (*p* < 0.05). It can be inferred that the use of compound prevention and control measures can effectively slow down the adaptation process of wild boar, leading to higher sustainability and a better effect of prevention and control measures. Unfortunately, this study did not determine the optimal combination of prevention and control measures (i.e., there was no significant difference between the prevention and control combinations). In future studies, more prevention and control measures should be introduced, such as the odor markers of wild boar’s natural enemies (e.g., Siberian tigers and wolves), and further combined prevention and control measures to explore more effective compound prevention and control measures.

In summary, in order to alleviate the conflict between humans and wild boars, we put forward the following suggestions: (1) widen and deepen the narrow landscape elements around farmland to prevent wild boars from entering farmland; (2) focus on the border area between forest and farmland; and (3) use compound prevention and control measures to improve their effectiveness and sustainability.

## 5. Conclusions

Landscape ecology provides a new perspective for analyzing the invasive behavior of wild boars into farmland. The results of this research show that the degree of landscape fragmentation, average patch area, coefficient of variation, forest edge density, and the presence of ditches have significant impacts on the activity patterns and invasive behaviors of wild boars. A smaller degree of fragmentation and a larger continuous forest or farmland area provide better hiding and foraging conditions for wild boars, thus increasing the risk of wild boars invading farmland. To reduce the invasion area, it is necessary to reduce fragmentation through rational land use planning, strengthen the protection of landscape diversity, and monitor and manage wild boar activities. Setting up electronic fences or strengthening patrols at agricultural and forestry borders, as well as improving the design of ditches—for example, through measures such as widening, deepening, or adding other physical barriers—can effectively prevent wild boar invasion. In addition, single prevention and control measures have limited effectiveness, while composite measures are more conducive to slowing down the adaptability of wild boars and improving the sustainability of prevention and control. Future research should explore combinations of different prevention and control measures in order to develop optimal strategies, thus alleviating the conflicts between humans and wild boars.

## Figures and Tables

**Table 1:** Establishment of measures for prevention and control of damage to farmland caused by wild boars. Combination of recording of human shouting, gong, and dog sounds, three in each plot; repellent 1: sulfur powder and lime powder; repellent 2: mint oil, pepper oil, and lard; warning lamp: red flashing solar warning lamps with installation height of 1.2 m or 2 m, respectively, four in each plot.

Number	Treatment Measure	Quantity	Area
A	Electronic fence with 2.0 m high warning light	3	0.5
B	Electronic fence with 1.2 m high warning light	3	0.5
C	Electronic fence with loudspeaker	3	0.5
D	Electronic fence with repellent 1	3	0.5
E	Electronic fence with repellent 2	3	0.5
F	Electronic fence with loudspeaker and 1.2 m warning light	3	0.5
G	Electronic fence with loudspeaker plus repellent 1	3	0.5
H	Electronic fence plus loudspeaker plus repellent 2	3	0.5
I	Electronic fence with 1.2 m warning light plus repellent 1	3	0.5
J	Electronic fence plus 1.2 m warning light plus repellent 2	3	0.5
K	Electronic fence	3	0.5
L	No measures	3	0.5

**Table 2:** Statistical test results of the optimal model for invaded farmland landscape characteristics. AREA_MN: mean patch area; AREA_CV: patch size coefficient of variation; FED: edge density of forest; RPLAND: percentage of residential area; SHEI: Shannon evenness index; FPLAND: percentage of forest; LED: edge density of farmland.

	Ref.df	Estimate	t Value	F	*p*
AREA_MN	/	0.022	2.849	/	0.005
AREA_CV	/	0.014	2.245	/	0.027
FED	/	0.110	2.283	/	0.025
s(RPLAND)	6.000	/	/	4.398	0.001
s(SHEI)	3.084	/	/	4.221	0.008
s(FPLAND)	6.618	/	/	2.249	0.024
s(LED)	3.478	/	/	2.886	0.023

**Table 3:** Statistical testing results of the optimal model for the invaded farmland characteristics. CANAL, canal; EF, electronic fence; DISCYS, distance from the village; DISWOOD, distance from the forest; PD, population density.

	Ref.df	Estimate	t Value	F	*p*
as.factor(CANAL)	/	0.759	2.136	/	0.035
as.factor(EF)	/	−0.927	−1.543	/	0.126
s(DISCYS)	3.959	/	/	4.472	0.003
s(DISWOOD)	1.991	/	/	5.320	0.009
s(PD)	4.698	/	/	4.032	0.002

**Table 4:** Prevention and control effects of different combinations of protective measures.

Number	Number of Invaded Plots	Invasion Times	Number of Wild Boars	Loss Rate
A	3	1.67 ± 0.58	2.00 ± 1.00	2 ± 1
B	3	1.00 ± 0.00	1.00 ± 0.00	1 ± 1
C	2	1.00 ± 0.58	1.67 ± 1.53	2 ± 2
D	2	1.67 ± 1.53	1.67 ± 2.08	6 ± 6
E	3	0.67 ± 0.58	3.33 ± 1.53	7 ± 4
F	2	2.67 ± 1.53	1.00 ± 1.00	1 ± 1
G	2	1.33 ± 1.53	1.33 ± 1.53	1 ± 1
H	2	0.67 ± 1.15	0.67 ± 1.15	0 ± 1
I	3	1.33 ± 0.58	2.33 ± 0.58	2 ± 1
J	2	1.00 ± 1.00	1.33 ± 1.53	1 ± 2
K	3	1.00 ± 0.58	7.33 ± 3.21	17 ± 13 *
L	3	4.67 ± 1.53	12.67 ± 4.04	54 ± 15 *

* Indicates a significant difference with respect to groups A–J (*p* < 0.05).

## Data Availability

Data are contained within the article.
